# A Sanatorium for Consumptive Children

**Published:** 1907-08-10

**Authors:** William Sinclair, Alfred Hoare, Edwin C. Bedford

**Affiliations:** (Archdeacon), Cawdor, M. Wellesley, Mary A. Ward; (Treasurer), Cawdor, M. Wellesley, Mary A. Ward; (Chairman), Cawdor, M. Wellesley, Mary A. Ward


### Editor's Letter-Box.

### A SANATORIUM FOR CONSUMPTIVE CHILDREN.

Sir,—In all great movements the hope of the reformer rests on the children, but we seem to be content to let consumptive children live or die unheeded. The longer we do so the greater grows the danger to the community, for every infected child is a centre of infection for others. The economic conditions which have led to the massing of populations in towns and cities may not be alterable, but we must make some effort before the health and vigour of our national life is sapped away by the grim and wide-spread pestilence of consumption. Every case must be grappled with individually. Each listless, suffering child, who watches with wistful eyes the sports it cannot share, must be taken away and placed in circumstances where special medical treatment, nourishing food, and pure air will give it a chance to become a healthy, vigorous man or woman.

The adult has been rather well cared for in the last few years. Large sanatoria have been completed, or put well on their way, at Midhurst, Northwood, Benenden, Frimley, and Davoz Platz (for the better-to-do), besides several smaller institutions. The children have been little thought of. In August, 1905, an appeal was put before your readers in support of the Children's Sanatorium (for the treatment of phthisis). Its progress has been small in the face of so much outlay needed for the adult. The start has, however, been made. In a new house adjoining the acquired site at Holt, in Norfolk, amongst pine woods and near the sea, provision is made for fifteen children, where, under the matron and visiting medical officer and the advice of an hon. consulting physician, we have with signal success for nearly twelve months received children. But this is all temporary until our funds are sufficient to justify a start in building. At present we want funds to continue to maintain the cases we can temporarily accommodate, and we want our building fund augmented to allow of starting in a permanent building. We have, since the commencement, gathered some £3,000; of which £1,000 is in hand for building, and £1,000, a gift for endowment, invested. The children of the well-to-do will be shortly going away for long holidays by sea and country side. Is it too much to expect that those who are able to bring health and happiness to their own or to have bright and healthy children around them will altogether forget those less fortunate who are left behind to waste away amid surroundings unhealthy, overcrowded, often hideous. Our work is in its infancy, when help is most needed. Truly, he gives twice who gives quickly. Donations and subscriptions may be paid to "The Children's Sanatorium Account," at Messrs. Hoare's Bank, 37 Fleet Street, or be sent to the Hon. Secretary, T. H. Wyatt, Esq., M.V.O., at the office, 68 Denison House, Vauxhall Bridge Road, S.W., by whom all particulars will be gladly supplied.

We are, faithfully yours,

Cawdor, William Sinclair (Archdeacon),

M. Wellesley, Alfred Hoare (Treasurer),

Mary A. Ward, Edwin C. Bedford (Chairman).

August 7, 1907.

THE HOSPITAL *August* 10, 1907.

